# Circadian Rhythm Dysregulation in Inflammatory Bowel Disease: Mechanisms and Chronotherapeutic Approaches

**DOI:** 10.3390/ijms26083724

**Published:** 2025-04-15

**Authors:** Yuko Nagao, Akihiko Taguchi, Yasuharu Ohta

**Affiliations:** 1 Health Science Center, Yamaguchi University, 1677-1 Yoshida, Yamaguchi 753-8511, Japan; 2 Department of Endocrinology, Metabolism, Hematological Science and Therapeutics, Graduate School of Medicine, Yamaguchi University, 1-1-1, Minami Kogushi, Ube 755-8505, Japan; yohta@yamaguchi-u.ac.jp

**Keywords:** circadian rhythm, clock gene, IBD, UC

## Abstract

Inflammatory bowel disease (IBD), comprising ulcerative colitis (UC) and Crohn’s disease (CD), is characterized by chronic intestinal inflammation. Recent research has highlighted the significant interplay between IBD pathogenesis and circadian rhythms. This review synthesizes current evidence regarding circadian regulation in IBD, covering three main areas: (1) circadian rhythms in intestinal physiology, (2) circadian disruption patterns in IBD patients, and (3) the role of clock genes in IBD pathogenesis. We discuss how these findings may inform novel chronotherapeutic approaches for IBD treatment. Future research directions that could facilitate translation of chronobiological insights into clinical applications are also explored.

## 1. Introduction

Circadian rhythms are endogenous autonomous mechanisms of physiological activity that result in a 24 h day/night cycle. In humans, the rhythm is set at 24 h 11 ± 16 min, slightly longer than the daily (24 h) rhythm, and can be synchronized by environmental factors such as light [1]. The center of circadian rhythms is the suprachiasmatic nucleus (SCN) of the hypothalamus, where the rhythm is generated by a set of genes known as clock genes. In recent years, the interaction between inflammatory bowel disease (IBD) and circadian rhythms has been widely reported, and their importance has attracted attention [2]. IBD is a chronic inflammatory disease, including ulcerative colitis (UC) and Crohn’s disease (CD), and multiple factors such as environmental and genetic parameters as well as immune system abnormalities have been implicated in the onset and exacerbation of IBD [3,4]. Notably, circadian rhythm disruption in patients with IBD is not merely an accompanying symptom but may be actively involved in the pathogenesis of the condition [5,6].

In this review, we focus on clock genes and output system genes in IBD and provide an overview of the latest research.

## 2. Circadian Rhythms

### 2.1. Molecular Mechanisms Underlying Circadian Rhythms

The core of the molecular mechanism of circadian rhythms is the transcription-translation feedback loop of clock genes [7,8], and the genes involved in this circuit are called “core clock genes” [9].

Specifically, the aryl hydrocarbon receptor nuclear translocator (ARNTL, also known as BMAL1), circadian locomotor output cycle kaput (CLOCK), periods (PER), and cryptochromes (CRY) form the core loop. In addition, there are several subloops that couple to the core loop, including a stabilizing loop that mainly consists of the nuclear receptor reverse erythroblastosis virus-α (REV-ERBα) and the retinoic acid-related orphan receptor (ROR)-response element (RORE) sequence, as well as a D-box loop in which D-box binding protein (DBP), E4 promoter binding protein 4 (E4BP4, also known as NFIL3), hepatic leukemia factor (HLF), and thyrotroph embryonic factor (TEF) bind to the D-box and function as output clock genes.

The heterodimer of CLOCK-BMAL1, a set of the core clock gene products, binds to the promoters of the *PER* and *CRY* clock genes, thereby activating both *PER* and *CRY* transcription [10]. The translated PER and CRY suppress CLOCK-BMAL1 transcription through a negative feedback mechanism, and this loop cycles once every 24 h to generate the circadian rhythm [11]. In addition, nuclear receptors such as REV-ERBα and RORα regulate transcription through the ROR/REV-ERB response element (RRE) present in transcriptional control regions such as the *BMAL1* gene, thereby reinforcing this feedback loop [12].

**Figure 1:**
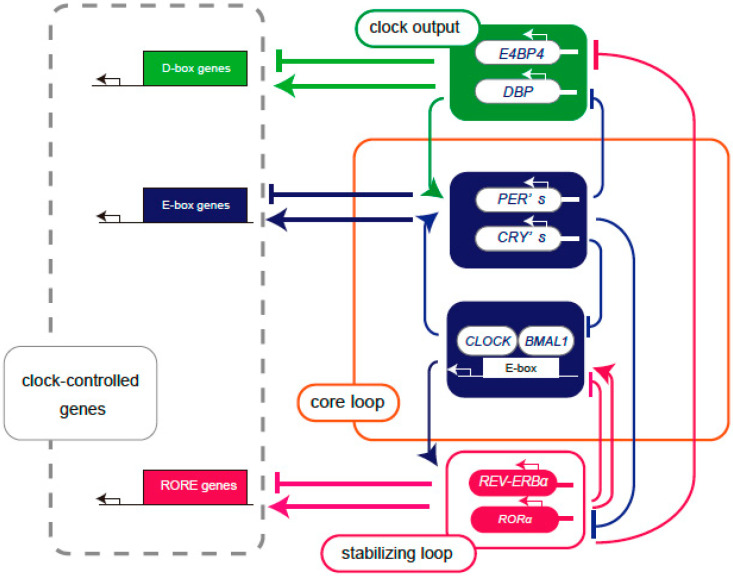
Schema of the feedback loop of the transcriptional regulation of clock genes. Core loop (orange square): CLOCK and BMAL1 proteins form a heterodimer and bind to the E-box, enhancing the expression of *PERs* and *CRYs*. Translated PERs and CRYs suppress their own gene expression by inhibiting CLOCK-BMAL1-mediated transcription. Stabilizing loop (red square): The RORE sequence contains REV-ERBα as a transcriptional repressor and RORα as a transcriptional activator and controls transcription through the clock cis elements E-box and RRE. Clock output (green background): DBP and E4BP4 bind to the D-box as a transcriptional activator and transcriptional repressor, respectively, to control downstream clock-controlled genes. The core loop signal controls the expression of *DBP*, and the stabilizing signal controls the expression of *E4BP4*. The core loop and multiple regulatory subloops are expressed at different phases of the circadian cycle of transcriptional activation, producing peaks of gene transcriptional activity at various times throughout the day.

Core clock genes, such as *BMAL1* and *CLOCK*, generate circadian rhythms by regulating a group of genes with E-box sequences that provide a rhythm underlying cellular functions. The output clock genes include *DBP*, *TEF*, *HLF*, and *E4BP4*, and the CLOCK- BMAL1 complex binds to the E-box in the promoter region of the *DBP* gene. The promoter region of the *DBP* gene is a CLOCK/BMAL1 binding region, known as a cis-regulatory element, referred to as the E-Box. The translated DBP binds to DNA recognition sites called the D-box and activates the transcription of downstream genes. The *E4BP4* promoter has an RRE region to which ROR and REV-ERB bind, and translated E4BP4 binds to the same D-Box as DBP [13] (Figure 1). Although *DBP* and *E4BP4* are not considered core clock genes for circadian rhythms, they may play a role in fine-tuning the core network, since the cycle length of circadian rhythms varies depending on the expression levels of DBP and E4BP4 [14]. Multiple regulatory subloops back up the oscillations of the core loop, generating peaks in gene transcriptional activity at different times of day [15].

### 2.2. Central and Peripheral Clock

The SCN functions as the master circadian clock. The circadian rhythm is modulated by environmental signals such as light, temperature, social interactions, and feeding patterns. The most important signal is light, which reaches the circadian system through retinal ganglion cells that contain the light-sensitive melanopsin [16,17]. In addition to light exposure, exogenous melatonin administration and physical activity can induce circadian phase shifts [16]. Rhythms in clock gene and protein expression have been observed in cells and tissues throughout the mammalian body, and these rhythms persist in culture, indicating that non-SCN cells also contain endogenous circadian oscillators [17]. The SCN acts as a “standard time regulator”, synchronizing peripheral tissue clocks [18]. For instance, in a study, when SCN-ablated hamsters received SCN transplants, behavioral rhythms were restored, and gene expression rhythms led to recovery in some peripheral tissues such as the liver and kidneys [19]. The central clock is primarily controlled by the brain’s SCN, which controls the circadian rhythms of several physiological and behavioral events through central nervous system neurons, including the paraventricular nucleus of the hypothalamus, which is important for neuroendocrine, homeostatic, and autonomic function integration [18].

However, circadian rhythms can also be mediated by “peripheral clocks” located in multiple tissues, including the intestines, liver, pancreas, and adipose tissue [20]. Feeding time serves as the main factor regulating peripheral clocks. When feeding is restricted to limited times in the light–dark cycle, peripheral clocks become disconnected from the brain’s master clock and synchronize according to feeding times and their autonomy [20]. Time-restricted feeding (TRF) can at least partially restore transcriptional and behavioral rhythms in mice with genetically disrupted circadian clocks [20]. Liver-specific deletion of the circadian clock gene (*Bmal1*) in mice attenuates glucose-regulated gene expression and impairs whole-body glucose homeostasis [21]. This demonstrates that peripheral clocks are essential for gene expression within tissues and overall tissue function. Peripheral clocks are synchronized to the SCN through neural and hormonal pathways [17]. The endocrine system is central in synchronizing the SCN and peripheral clocks [22]. In summary, central and peripheral clocks influence each other through various factors and are synchronized and regulated by external factors.

### 2.3. Circadian Rhythms and Immune Function

Immune dysfunction is among the most important pathogenic features of many chronic diseases, including IBD. Immune cell numbers and functions exhibit diurnal variations, resulting in time-dependent changes in immune response efficiency. Neutrophil mobilization from the bone marrow shows marked circadian rhythms, with relatively low neutrophil numbers in the blood during the day, significantly increasing at the beginning of the night activity phase, reaching peak numbers [23,24]. This rhythmic mobilization of neutrophils from the bone marrow into the blood is regulated by adrenergic neural transmission and circadian expression changes in the bone marrow stromal cell chemokine CXCL12 [24]. Similarly, lymphocytes exhibit significant circadian rhythms in their flow and quantity variations within lymph nodes; during the daytime, lymph nodes contain relatively fewer lymphocytes, but when the nighttime active phase begins, a large number of lymphocytes rapidly enter the lymph nodes, causing rapid accumulation [23,25]. Interestingly, immunizations or exposure to vaccines during the time window when lymphocyte numbers peak daily can induce stronger immune responses [25,26]. Innate immune recognition pathways also exhibit distinct circadian rhythm patterns. For instance, Toll-like receptor 9 (TLR9) gene expression and the production of mRNA and protein peak in the early active period, resulting in rhythmic changes in its ability to recognize pathogens due to the receptor’s own daily fluctuations in expression [27].

The immune system is largely controlled by circadian rhythms, with immune cell activity, gene expression, and cytokine production showing circadian variations throughout the day [28]. NFIL3 (E4BP4) is necessary for innate lymphoid cell (ILC) development, including natural killer cells; similarly, B cell development depends on BMAL1 [29]. Clock genes are important in regulating immune cell function and inflammatory responses, contributing to maintaining immune system homeostasis in the body [28]. Other research has shown that clock proteins control fundamental aspects of immune responses [30]. For example, the CLOCK-BMAL1 heterodimer directly controls the expression of TLR9, a pattern recognition receptor that recognizes bacterial and viral DNA, and suppresses inflammatory monocyte chemokine ligand (CCL2) expression. In addition, REV-ERBα suppresses IL-6 induction [30]. The molecular clock functions as a “controller” of immune cells, regulating immune responses depending on the integration of various inputs, including neural, hormonal, and local factors, and circadian rhythms in cellular gene expression [30].

The SCN clock harmonizes peripheral clocks through the hypothalamic-pituitary-adrenal (HPA) axis and autonomic nervous system and their respective hormones, glucocorticoids, and catecholamines (epinephrine and norepinephrine), functioning as synchronization messengers or “zeitgebers” for peripheral clocks [30]. Other hormones, such as glucocorticoids, catecholamines, prolactin, melatonin, and growth hormones, affect the immune system and peak at specific times of the day. This SCN control over autonomic and endocrine outputs enables the synchronization of peripheral clocks, including immune cells, allowing the coordination of temporal physiological programs across many tissues at specific times of day [30]. The central clock in the SCN and its rhythmic control over autonomic and endocrine systems are important determinants of immune regulation. Circadian rhythms divide the immune system into two states: one where animals prepare for activity and heightened alertness when infection and injury risks are high, requiring increased white blood cell counts and immune cell sensitivity, and another state occurring during rest periods, providing opportunities for inflammation suppression and tissue repair [30]. Circadian rhythms influence innate immunity and adaptive immune responses. These are regulated through complex mechanisms, and circadian rhythm disruption is involved in autoimmune diseases, cancer, and inflammatory diseases [28].

## 3. IBD

IBD primarily includes CD and UC [3,4]. CD can cause inflammation anywhere in the digestive tract, while UC primarily affects the colon. The exact pathogenesis of IBD is not well understood, with several factors presumed to influence its onset, including bacterial infections, immune system changes, and genetic mutations [4]. Beyond genetic predisposition, environmental factors such as diet and smoking also significantly impact the disease [31]. The prevalence of IBD is the highest in North America and Europe, affecting 0.3–0.6% of the population [32]. However, recent data suggest rapid increases in IBD prevalence in other regions, including Asia, Africa, and South America [33]. While it can develop at any age, onset is most common between ages 15 and 30, with a lower peak between 50 and 70 years [34].

### 3.1. Symptoms and Clinical Features

IBD symptoms vary depending on inflammation severity and location. Symptoms range from mild to severe and alternate between active and remission periods. Common symptoms in both UC and CD include abdominal pain and/or diarrhea (often with blood or mucus). These symptoms can fluctuate and may persist for weeks to months, unlike infectious gastroenteritis symptoms, which typically last only days [35]. Unlike functional gastrointestinal disorders where symptoms appear only during the day, IBD symptoms may continue at night. Additional symptoms may include loss of appetite, weight loss, extreme fatigue, and fever [35].

UC consistently shows mucosal inflammation causing edema, ulceration, bleeding, and electrolyte leakage. UC inflammation typically begins in the rectum and extends continuously to the proximal colon [3]. CD features skip lesions and can affect any part of the digestive tract, potentially causing strictures, inflammation, and fistulas. While UC’s continuous inflammation is limited to the mucosa and submucosa, CD affects the entire thickness of the digestive tract mucosa [3].

Common complications of IBD include colon cancer, intestinal perforation, fistulas, pelvic or perirectal abscesses, toxic megacolon, and extraintestinal manifestations, such as osteoporosis, deep vein thrombosis, anemia, gallstones, primary sclerosing cholangitis, arthritis, iritis, and skin lesions. Additionally, psychiatric illness rates are higher, including depression, suicidal tendencies, and anxiety [3].

### 3.2. Diagnosis and Evaluation

IBD diagnosis involves clinical findings, blood tests, stool tests, imaging (CT scans or MRI), and endoscopic biopsies [35]. The Crohn’s Disease Activity Index (CDAI) is the most commonly used scale for disease activity assessment for CD. This index scores factors including abdominal pain frequency, diarrhea episodes, weight changes, and general condition. The Harvey Bradshaw Index (HBI) is also used for CD. Similarly, UC uses measures such as the Mayo Score/DAI [3].

### 3.3. Treatment

IBD treatment aims to induce and maintain remission [3,35]. Drug therapy is used to improve symptoms and control mucosal inflammation. Early remission induction is desirable to minimize intestinal damage and should be achieved within three months [35].

UC treatment is tailored based on severity, extent, patient age, comorbidities, drug safety and efficacy, patient preferences, administration route, urgency, cost, extraintestinal manifestations (EIMs), and specific factors such as pregnancy, surgery, and pediatric populations [36].

Various treatment options are available for mild to moderate UC, including 5-aminosalicylates, corticosteroids, and immunosuppressants such as thiopurines and methotrexate [37]. For moderate to severe UC, treatment approaches shift to advanced biologics, such as anti-TNFα, anti-α4β integrin biological drugs, anti-interleukin 12/23, selective IL-23 inhibitors, and small molecule drugs like Janus kinase (JAK) inhibitors and sphingosine-1-phosphate (S1P) modulators [37]. Surgical treatment, such as total colectomy, may be necessary for treatment-resistant cases [3].

CD treatment varies depending on the affected digestive tract portions, degree of fistulas or strictures, and extraintestinal complications [3]. Mild ileocecal disease typically starts with mesalamine treatment and is intensified with budesonide, a steroid that undergoes first-pass metabolism in the liver to prevent systemic side effects. More extensive cases require systemic steroid therapy with prednisone. The goal is steroid withdrawal within six weeks; patients unable to achieve this may need additional immunomodulators such as 6-mercaptopurine, azathioprine, or low-dose methotrexate [3]. Moderate to severe cases may need anti-TNFα agents. Patients with severe fistulas may require surgical treatment.

### 3.4. Management

Both diseases require attention to nutritional status, mental health, bone health, vaccinations against preventable diseases, and cancer screening [3]. Patients with IBD should have their iron, vitamin B12, folate, and vitamin D levels measured every six months and take recommended supplements in case of deficiencies. IBD symptoms significantly impact psychological well-being and quality of life. Anxiety and depression are common in patients with IBD compared to the general population, making screening and psychiatric referrals important [35]. Patients with IBD have approximately 1.5–5 times higher mortality rates compared to the general population. Major causes of death include infections, disease progression, surgical complications, and multi-organ involvement. Importantly, due to higher colorectal cancer rates, colonoscopy screening is recommended every 1–2 years [3].

## 4. The Relationship Between the Intestine and Circadian Rhythm

### 4.1. Circadian Rhythm Regulation in the Intestinal Tract

The intestinal tissue has its own peripheral clock, which controls the circadian rhythm of various physiological functions, including immune response, intestinal barrier function, and nutrient absorption [6]. This peripheral clock system is a dynamic system that is entrained by neural and humoral signals from the central clock and influenced by changes in meal timing and gut microbiota [6]. Of particular interest, the gut microbiota itself is regulated by gender and the host circadian clock, suggesting that there is a bidirectional interaction between the gut microbiota and the host’s clock system [38]. Analysis of the fecal microbiota of mice in a previous study showed circadian rhythms in the absolute amount of fecal bacteria and the abundance of bacteroides, which were more pronounced in female mice [27]. When the host circadian clock was disrupted by the deletion of *Bmal1*, the rhythmicity in the fecal microbiota composition disappeared in both genders, and interaction was observed [38]. Furthermore, changes in the gut microbiota induced by a high-fat diet drive the reprogramming of liver circadian rhythms through PPARγ-mediated transcriptional activation in mice, suggesting that the intestinal peripheral clock also plays an important role in regulating the body’s metabolism [39].

The expression of clock genes in intestinal tissue has been confirmed in various cell types. The intestinal epithelial cells either continue to proliferate or cease dividing and differentiate depending on cell cycle regulation. Crosstalk may occur between circadian rhythms and molecules that control cell cycle progress, and several reports have linked cell proliferation in the gastrointestinal epithelium to the circadian clock [40]. Although the detailed mechanism explaining the temporal regulation is unclear, one possible mechanism is the direct regulation of key cell cycle regulators through transcriptional regulation via the circadian core clock loop. Another possible mechanism is the regulation of the proliferation rhythm by extrinsic luminal signals, neural output signals, gut intestinal hormones, and growth factors [40].

The innate immunity in the gut utilizes a host of antimicrobial polypeptides known as defensins to combat ingested bacteria [41]. The mouse intestinal defensins (cryptdins) are constitutively produced and secreted but are overexpressed during infection and inflammation. Under healthy conditions, the expression of cryptdin in the mouse intestine shows a circadian rhythm, suggesting that defensin expression in humans also peaks during the day [41].

Tight junction proteins, occludin and claudin-1, are important in regulating intestinal epithelial permeability. In mice, the expression of occludin and claudin-1 shows circadian oscillation opposite to that of the *Per2* mRNA level, and CLOCK and BMAL1 directly bind to the E-box of occludin and claudin-1 promoters and increase the transcriptional response [42]. Mice with a mutated *Per2* gene(m*Per2*^m/m^) showed constitutively high levels of occludin and claudin-1 proteins and were more resistant to dextran sulfate sodium (DSS)-induced colitis, whereas *Clock*^Δ19/Δ19^ mice showed decreased levels of these tight junction proteins and were more susceptible to DSS-induced colitis [42]. These results suggest that the expression of the tight junction proteins occludin and claudin-1 is under the control of circadian rhythms and may affect the diurnal variation of intestinal permeability and the susceptibility to colitis.

The peripheral clock is important for nutrient absorption and metabolism. The expression and activity of H(+)/peptide cotransporter 1 (PEPT1) in intestinal epithelial cells is important for the transport of nutrients and drugs. Its expression and activity show circadian rhythms, which regulate the efficiency of nutrient absorption in a time-dependent manner [43]. In animal models, the expression of DBP was synchronized with PEPT1, and DBP directly induced the transcriptional activity of PEPT1, indicating that DBP plays a crucial role in the circadian oscillation of PEPT1 [43]. Interestingly, some aspects of circadian rhythmicity in small intestinal function, such as nutrient transport and enzyme activities, persist even during intravenous feeding that bypasses the intestine, suggesting regulation by neuroendocrine mechanisms rather than direct nutrient sensing [44].

These findings indicate that the peripheral clock system in the intestinal tract regulates three major functions in an integrated manner: immune response, barrier function, and nutrient absorption. More importantly, these functions are closely interrelated, and the disruption of one function may affect the others. The integrated breakdown of these functions may play a role in the pathogenesis of IBD.

### 4.2. Altered Clock Gene Expression in Patients with IBD

Alterations in clock gene expression in patients with IBD have been reported by several research groups. IBD is a multifactorial disease that causes an abnormal immune response in genetically susceptible individuals and results from a complex interrelationship between environmental/microbial factors and the intestinal immune system. In CD and UC patients, genetic polymorphisms in the clock gene PER3 were evaluated, and the rs2797685 variant was significantly increased in both CD (*p* = 1.6 × 10^−4^, odds ratio [OR] = 1.38, 95% confidence interval [CI]: 1.17–1.63) and UC (*p* = 0.12, OR = 1.25, 95% CI: 1.05–1.48) patient groups compared to healthy controls [45]. A study by Liu et al. showed that the expression rhythms of key clock genes, including *BMAL1*, *CLOCK*, *PER1*, *PER2*, *PER3*, *CRY1*, and *CRY2*, were significantly disrupted in the colon mucosa of patients with active IBD. In particular, the expression of *BMAL1* and *PER2* was significantly decreased by up to one third, and these changes correlated with increases in inflammatory cytokines [5]. Furthermore, the expression of *BMAL1*, *CRY1/2*, and *REV-ERBα* decreased by up to one half in patients with IBD and sleep disorders, and sleep quality was correlated with disease severity in these patients [46].

Another study analyzed clock gene expression in endoscopic mucosal biopsies from IBD patients and found that *ARNTL2* and *RORα* were elevated in IBD patients, whereas *CSNK2B*, *NPAS2*, *PER1*, and *PER3* were decreased in CD patients [47]. On the other hand, *ARNTL2*, *CRY1*, *CSNK1E*, and *TIPIN* were elevated in UC patients, whereas *NR1D2* and *PER3* were decreased, indicating differences in clock gene expression between CD and UC [47].

Clock gene expression is decreased in patients with active UC. However, recent studies have demonstrated that the expression of clock genes, such as *BMAL1*, *CLOCK*, *PER1*, and *CRY1*, is significantly increased in the colonic mucosa of children with active UC, whereas the expression of *PER2* is decreased [48]. Although this report differs from previous reports, patients with active IBD show great variability in gene expression [5,49]. Interestingly, a uniform pattern of gene expression was found in healthy controls compared to the highly variable expression pattern in patients with UC. Among the healthy controls, inflammatory genes (TNFα, IL10, IL6, NFκB) were positively correlated with clock genes (*BMAL1*, *CLOCK*, *PER1*, *PER2*, *CRY1*, *CRY2*) [48].

Clock genes crucially regulate the intestinal immune system. Recent studies have demonstrated that PER2 negatively regulates CD4+ T cell IFN-γ production in UC [50]. PER2 reduces the binding activity of ADAMS12, a T cell costimulatory molecule that contributes to tissue inflammation, and suppresses IFN-γ production by CD4+ T cells. In patients with IBD, decreased expression of PER2 in the intestinal mucosa may promote intestinal inflammation through enhanced IFN-γ production [50]. Interestingly, this pathogenic role of decreased PER2 in human IBD appears to contrast with findings in mouse models [42], where mice with a mutated *Per2* gene showed increased expression of tight junction proteins (occludin and claudin-1) and decreased susceptibility to DSS-induced colitis. These seemingly contradictory findings suggest that PER2 may have distinct roles in different aspects of intestinal homeostasis: immune regulation and barrier function.

When comparing clock protein expression in the intestinal mucosal epithelial cells of 24 patients with CD and 26 patients with UC and controls, the expressions of BMAL1, PER1, PER3, TIMELESS, and NPAS2 decreased in patients with IBD, while no significant differences were observed in BMAL2, CLOCK, and PER2 expression [51]. Although the findings regarding *PER2* expression levels appear inconsistent with its functional roles discussed above, PER2 remains a potential therapeutic target for IBD that merits further investigation.

In contrast, the role of BMAL1 in intestinal homeostasis is more clearly defined. Decreased expression of Bmal1 due to disruption of the circadian rhythm leads to decreased levels of tight junction proteins and increased apoptosis of intestinal epithelial cells, thereby impairing intestinal barrier function. Studies using DSS-induced colitis models in mice have demonstrated that decreased Bmal1 expression exacerbates inflammation through impaired intestinal barrier function [42,52].

Supplementation therapy with butyrate, a short-chain fatty acid, may contribute to the normalization of clock gene expression in patients with IBD. A double-blind randomized controlled trial showed that butyrate supplementation increased the expression of *CRY1/2*, *PER1*, and *BMAL1* in patients with active UC while simultaneously reducing the biomarkers of inflammation and improving sleep quality and quality of life [53].

These findings suggest that abnormalities in clock gene expression play an important role in the pathophysiology of IBD and that correcting these abnormalities may be a promising new therapeutic strategy. In particular, the importance of the control of the intestinal immune system through the regulation of clock gene expression, as well as sleep and circadian rhythms, is emphasized.

### 4.3. The Role of E4BP4 in IBD Pathogenesis

Output system clock genes are a group of downstream genes controlled by core clock genes. Several reports have described the relationships between output clock genes and metabolic regulation [13], but reports on their relationship with immune function are scarce. E4BP4 is essential for the development of NK cells and dendritic cells and is also involved in macrophage activation and the polarization of CD4+ T cell responses [54,55]. E4BP4 is widely distributed in immune cells and is an important transcription factor in maintaining intestinal immune homeostasis. Dysfunctional E4BP4 is involved in the pathogenesis of IBD [55].

In cultured macrophages and in vivo models, E4BP4 expression is increased upon exposure to microorganisms. The expression of E4BP4 is reduced in CD14-positive lamina propria mononuclear cells from patients with CD and UC compared with healthy controls. The cytokines IL-12 and IL-23, which are important in the pathogenesis of IBD, are mainly produced by macrophages and dendritic cells [55]. The two cytokines form heterodimeric proteins that share a common subunit, p40, and E4BP4 is involved in the development of IBD by negatively regulating the secretion of IL-12p40 in the intestine. Indeed, E4BP4 knockout mice spontaneously develop colitis and rectal prolapse, which are associated with the overproduction of IL-12p40 in the serum and colon [56]. E4BP4 is induced by IL-10 and serves as an IL-12p40 inhibitor in macrophages upon encounter with bacterial products [56]. E4BP4 and IL-10 double-knockout mice develop severe early-onset colitis and show upregulated IL-12p40 expression [55]. Genetic deletion of *Il12b* in E4BP4 knockout mice completely prevents the development of colitis. These results suggest that E4BP4 and IL-10 are involved in intestinal mucosal homeostasis and dysregulation of IL-12b is involved in the development of colitis [55]. Furthermore, E4BP4 knockout mice are more susceptible to colitis when housed in conventional environments, indicating that the colitis development in E4BP4-deficient mice is dependent on the gut microbiota [56]. E4BP4 in macrophages induces an anti-inflammatory phenotype that ameliorates the severity of DSS-induced colitis [57]. E4BP4 can bind to the *Il4rα* gene, which promotes anti-inflammatory properties, and may contribute to increased anti-inflammatory gene expression. Not only the core clock genes but also E4BP4, one of the output clock genes, may be potentially important factors regulating gut immunity. E4BP4 is a promising new therapeutic target for IBD.

## 5. Treatment Strategies Targeting the Circadian Rhythm

Therapeutic approaches targeting the regulation of circadian rhythms have been attracting attention as a new treatment strategy for IBD. Incorporating a chronobiological perspective into existing treatments can optimize their effectiveness.

Light therapy is expected to be effective. The effectiveness of morning light therapy has been reported for fibromyalgia, a condition that causes chronic pain [58]. Given that patients with IBD have disrupted circadian rhythms, and light stimulation can improve circadian rhythms, morning light therapy is expected to improve quality of life and disease activity. Therefore, clinical trials have been initiated to investigate the effectiveness of morning light therapy [59].

Diet modulation represents another promising approach for targeting circadian rhythms in IBD. Intermittent fasting (IF), including TRF, has been shown to influence circadian rhythms by serving as a dominant Zeitgeber that can reset the circadian clock [60]. The molecular mechanisms behind this effect involve alterations in metabolic factors such as NAD+, sirtuins, and AMP-activated protein kinase (AMPK), which are modified during fasting periods and directly influence circadian rhythm regulation [61,62,63,64]. Studies have demonstrated that fasting improves the amplitude and stability of circadian rhythms while synchronizing oscillation phases, which is particularly relevant for IBD patients who often exhibit disrupted circadian rhythms that increase disease susceptibility and severity [2].

In animal models of colitis, various fasting protocols have shown promising results. Time-restricted feeding significantly reduced inflammatory markers, including TNF-α, IL-1β, and IL-6, in colitis models [65,66]. Moreover, TRF improved intestinal barrier function by increasing the expression of tight junction proteins such as Claudin-1, ZO-1, and occludin [66]. Importantly, TRF also favorably modulated the gut microbiota, suppressing colitis-associated bacteria while enhancing the abundance of beneficial short-chain fatty acid-producing microbes such as Rikenellaceae, Lactobacillus, and Ruminococcus [66]. These findings are particularly relevant, as microbiota disruption is a key factor in IBD pathogenesis.

Studies in healthy humans have shown that time-restricted eating (TRE) significantly reduces inflammatory markers, including IGF-1, IL-6, TNF-α, and IL-1β [67,68]. Additionally, TRE increases the expression of *BMAL-1* and *CLOCK*, as well as *SIRT1* gene expression [69], further supporting the potential chronobiological benefits of dietary timing interventions. While comprehensive clinical studies in IBD patients are still needed, preliminary evidence from Ramadan fasting studies suggests that controlled fasting can be well-tolerated by most IBD patients [70]. These findings collectively suggest that incorporating carefully designed dietary timing strategies may offer a non-pharmacological approach to restore circadian rhythm function and potentially improve inflammatory outcomes in IBD patients [71].

As a complementary approach, administration of butyrate, a short-chain fatty acid, restored clock gene expression and improved sleep quality [53]. Urolithin A (UA), an intestinal microbial metabolite, is important in improving the intestinal barrier function and circadian rhythm [72]. Administration of UA to mice with a DSS-induced colitis model improved the expression of tight junction associated genes (*Cldn1* and *Cldn4*) and clock genes (*Bmal1* and *Per2*) in the colon [72]. Similarly, in the SCN, *Bmal1* and *Per2* showed 24-h expression oscillations similar to those seen in colon samples, and gene expression was upregulated by the administration of UA [72]. Therefore, UA supplementation improves irregular circadian rhythms in both intestinal barrier function and the SCN, demonstrating its potential to improve IBD associated with sleep disorders.

Development of therapeutic drugs targeting clock genes is also underway. The nuclear receptors *REV-ERB* (*REV-ERBα*, *REV-ERBβ*) and *RORs* (*RORα*, *RORβ*, and *RORγ*) are involved in many physiological processes, including metabolism, development, immune regulation, and circadian rhythm, and may serve as therapeutic targets for treating several diseases [73]. Circadian rhythm modulators, such as REV-ERB agonists and RORγt inverse agonists, are expected to be novel therapeutic agents for treating IBD [74,75]. The RORγt inverse agonist TAK-828F suppresses the progression of colitis in anti-TNFα non-responsive colitis model mice and dose-dependently reduced Th17 cells in mesenteric lymph nodes. In normal mice, administration of TAK-828F does not reduce peripheral and intestinal lymphocyte counts, as observed in *RORγ* knockout mice [75,76]. These agents can modulate clock gene expression and immune response and may be an effective therapeutic approach against IBD [74,75].

## 6. Conclusions

In this review, we focus on clock genes in IBD and describe recent results. Circadian rhythms and IBD are closely related, but the relationship is complex and multifactorial, and the detailed mechanisms have not been fully elucidated.

In the future, it is necessary to explore the relationships between intestinal immunity, intestinal microbiota, the central clock, and the peripheral clock in the intestine. Further research into the individual interactions and the resulting overall control system may not only deepen our understanding of the pathogenesis of IBD but may also lead to the development of new treatment strategies. In the long term, it will be necessary to evaluate the effectiveness and safety of therapeutic interventions based on chronobiology, and research is expected to result in progress in translating basic research findings into clinical applications.

## Data Availability

This is a review article and does not contain any new data. All information discussed is based on previously published studies that are cited within the text.

## Abbreviations

AMPK	AMP-activated protein kinase
ARNTL	aryl hydrocarbon receptor nuclear translocator like, known as BMAL1
BMAL1	brain and muscle Arnt-like protein 1
CD	Crohn’s disease
CDAI	Crohn’s Disease Activity Index
CLOCK	circadian locomotor output cycles kaput, paralogous to NPAS2
CRY	cryptochromes
DBP	D-box binding protein
DSS	dextran sulfate sodium
E4BP4	E4 promoter-binding protein 4
EIMs	extraintestinal manifestations
HBI	Harvey Bradshaw Index
HLF	hepatic leukemia factor
HPA	hypothalamic-pituitary-adrenal
IBD	inflammatory bowel disease
IF	intermittent fasting
ILC	innate lymphoid cell
JAK	Janus kinase
NFIL3	nuclear factor, interleukin 3 regulated protein, known as E4BP4
NR1D1	nuclear receptor subfamily 1 group D member 1
PEPT1	peptide cotransporter 1
PER	periods
REV-ERBα	reverse erythroblastosis virus-α, known as NR1D1
RRE	ROR/REV-ERB response element
ROR	retinoic acid receptor-related orphan receptor
SCN	suprachiasmatic nucleus
TEF	thyrotroph embryonic factor
TLR 9	Toll-like receptor 9
TRE	time-restricted eating
TRF	time-restricted feeding
UA	Urolithin A
UC	ulcerative colitis
